# A high resolution RH map of the bovine major histocompatibility complex

**DOI:** 10.1186/1471-2164-10-182

**Published:** 2009-04-24

**Authors:** Candice L Brinkmeyer-Langford, Christopher P Childers, Krista L Fritz, Ashley L Gustafson-Seabury, Marian Cothran, Terje Raudsepp, James E Womack, Loren C Skow

**Affiliations:** 1 Department of Veterinary Integrative Biosciences, College of Veterinary Medicine, Texas A&M University, College Station, Texas 77843-4458, USA; 2 Department of Biology, Georgetown University, Washington, DC 20057, USA; 3 Department of Veterinary Pathobiology, College of Veterinary Medicine, Texas A&M University, College Station, Texas 77843, USA

## Abstract

**Background:**

The cattle MHC is termed the bovine leukocyte antigen (BoLA) and, along with the MHCs of other ruminants, is unique in its genomic organization. Consequently, correct and reliable gene maps and sequence information are critical to the study of the BoLA region. The bovine genome sequencing project has produced two assemblies (Btau_3.1 and 4.0) that differ substantially from each other and from conventional gene maps in the BoLA region. To independently compare the accuracies of the different sequence assemblies, we have generated a high resolution map of BoLA using a 12,000_rad _radiation hybrid panel. Seventy-seven unique sequence tagged site (STS) markers chosen at approximately 50 kb intervals from the Btau 2.0 assembly and spanning the IIa-III-I and IIb regions of the bovine MHC were mapped on a 12,000_rad _bovine radiation hybrid (RH) panel to evaluate the different assemblies of the bovine genome sequence.

**Results:**

Analysis of the data generated a high resolution RH map of BoLA that was significantly different from the Btau_3.1 assembly of the bovine genome but in good agreement with the Btau_4.0 assembly. Of the few discordancies between the RH map and Btau_4.0, most could be attributed to closely spaced markers that could not be precisely ordered in the RH panel. One probable incorrectly-assembled sequence and three missing sequences were noted in the Btau_4.0 assembly. The RH map of BoLA is also highly concordant with the sequence-based map of HLA (NCBI build 36) when reordered to account for the ancestral inversion in the ruminant MHC.

**Conclusion:**

These results strongly suggest that studies using Btau_3.1 for analyses of the BoLA region should be reevaluated in light of the Btau_4.0 assembly and indicate that additional research is needed to produce a complete assembly of the BoLA genomic sequences.

## Background

The typical mammalian major histocompatiblity complex (MHC) contains a cohort of closely linked and highly polymorphic genes and gene families, many of which participate in immunity [1]. These genes are usually organized into a tightly linked complex defined by three regions or classes. Class I molecules are ubiquitously expressed on nucleated cells and function to present endogenous peptides to CD8+ T cells. Class II molecules are expressed exclusively on antigen-presenting cells including macrophages, dendritic cells and B lymphocytes, and present peptides of exogenous origins to CD4+ helper T cells. Loci in the class III region encode a diverse set of proteins, including many cytokines, but not all genes in the class III region are involved in immunity.

The cattle MHC, termed the bovine leukocyte antigen (BoLA), is similar to the MHCs of other species in that genes within BoLA encode proteins that participate in the adaptive and innate immune responses [2] and play crucial roles in determining host response to pathogens. However, the organizational features of the MHCs of cattle and other ruminants are unique in that class II genes occur in two segments rather than a single segment as observed in other mammalian species (e.g. human [3], mouse [4], dog [5], and horse [6]). The two segments are located about 20 cM apart and are designated class IIa and class IIb [7-9]. Class IIa is closely associated with the class I and class III regions, while class IIb is positioned closer to the centromere. The unique separation of class II loci, of related function and tightly linked in other species, makes the study of this part of the bovine genome a high priority for understanding the processes involved in coordinated gene regulation, structure and evolution of the MHC.

Linkage analyses (e.g., [10-12]) and physical gene maps [8,13-16] have defined the general organization of BoLA, but do not provide the detail provided by a sequence-based map. Results from the bovine genome sequencing project have produced a preliminary assembly 2.0 and two subsequent assemblies (Btau_3.1 and 4.0) that differ considerably from each other and from conventional gene maps [16,17]. The most recent sequence assembly, Btau_4.0, incorporated additional mapping information [14], fingerprint contig (FPC) maps, and bovine and sheep BAC end sequences [18] to resolve many of the inconsistencies of the two prior genome assemblies but has not been independently verified.

To compare the accuracies of the 3.1 and 4.0 sequence assemblies of the bovine MHC, we generated a high resolution map of BoLA using a 12,000_rad _radiation hybrid panel [19]. The resolution achievable using this panel exceeds that of the 5,000_rad _panel [20] previously used to generate medium-density maps for cattle chromosomes (e.g., [8,21-25]) due to the increased frequency of radiation-induced chromosomal breaks. This makes the 12,000_rad _panel suitable for fine mapping genomic regions of interest (e.g., [26]). The map described here documents assembly errors in the Btau_3.1 assembly and largely validates the revisions contained in the 4.0 assembly. We also compared the RH map of BoLA with the version 36 assembly of HLA to demonstrate overall conservation of gene order between BoLA and HLA and to further validate the hypothesis that a single ancestral inversion accounts for the organizational differences between the bovine and human MHCs. This information is critical for identifying linkage disequilibrium and haplotype structure of the BoLA region and to facilitate accurate comparative genomic studies of BoLA and the MHCs of other species.

## Results

### Marker development

Seventy-seven of the original 80 primer pairs were used for mapping. Fourteen of the 77 markers were located in the class I and class I extended regions of BoLA, 27 were located within class IIa or IIa extended regions, 17 were located within class IIb, and 19 were located within class III (more details about marker class and position is available in Additional file 1). Of the three markers not retained for mapping, one marker (12.00) was dropped from analysis due to typing ambiguities and two markers (48.10 and 55.30) could not be assigned to specific locations and instead were binned within the IIa/III/I group. Thirty-one of the 184 clones in the RH panel consistently gave ambiguous genotyping results (typed as "2" for two or more markers) and were excluded from computation of the RH maps.

### RH mapping

Analysis of the distribution of STS markers across the RH panel resulted in the localization of markers into two groups, corresponding to the BoLA IIb region and the BoLA IIa, III, and I region. The IIb group of 17 markers was distributed along 125.6 cR, for a resolution of 1 marker/7.4 cR. The IIa/III/I group of 60 markers was distributed over 496.6 cR at a resolution of 1 marker/8.3 cR. Resolution in kb was also determined by dividing by the size in base pairs of each region in Btau_4.0 by the number of markers with unique vectors (clustered markers counted as single markers). Using this method, resolution of the RH group for the IIb region of BoLA is approximately one marker every ~221 kb and resolution for the IIa/III/I region of BoLA is one marker every ~184 kb.

**Figure 1:**
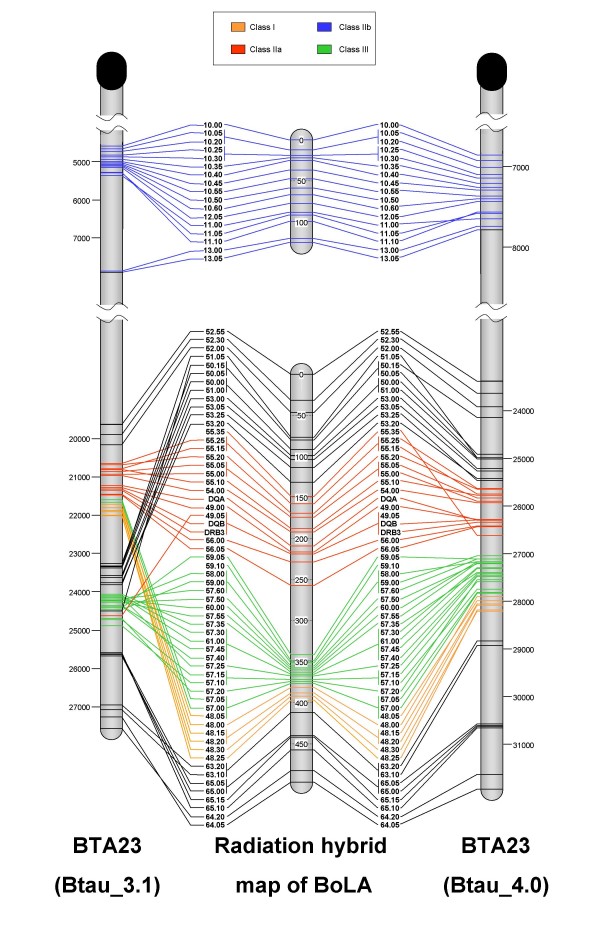
**High-resolution radiation hybrid and comparative maps of BoLA**. To the far left and far right are representations of BTA23 generated using the current genome sequence assemblies (Btau_3.1 and Btau_4.0, respectively). The centromere is represented as a black oval and distances are given in kilobases (Kb). The radiation hybrid map of BoLA is in the center, with map units given as centirays (cR). Just to the left and right of the RH map are marker names given in the order ascertained via radiation hybrid analysis, with connecting lines illustrating their comparative locations on the BTA23 maps.

Twelve (71%) of the markers in the IIb group and 26 (43%) of the markers in the IIa/III/I group gave unique RH vectors and were designated as MLE-consensus (frame) and ordered with highest confidence. Positions, lod scores, and frame/placed status of individual markers are presented in Additional file 2. Retention frequencies of markers in the IIb group ranged from 12.2% to 15.7%, with an average value of 14.3%. For the IIa/III/I group, retention frequencies ranged from 13.9% to 27.3%, with an average value of 21.1%. RH maps developed for the two BoLA regions are presented in Figure 1.

### Comparative maps

**Figure 2:**
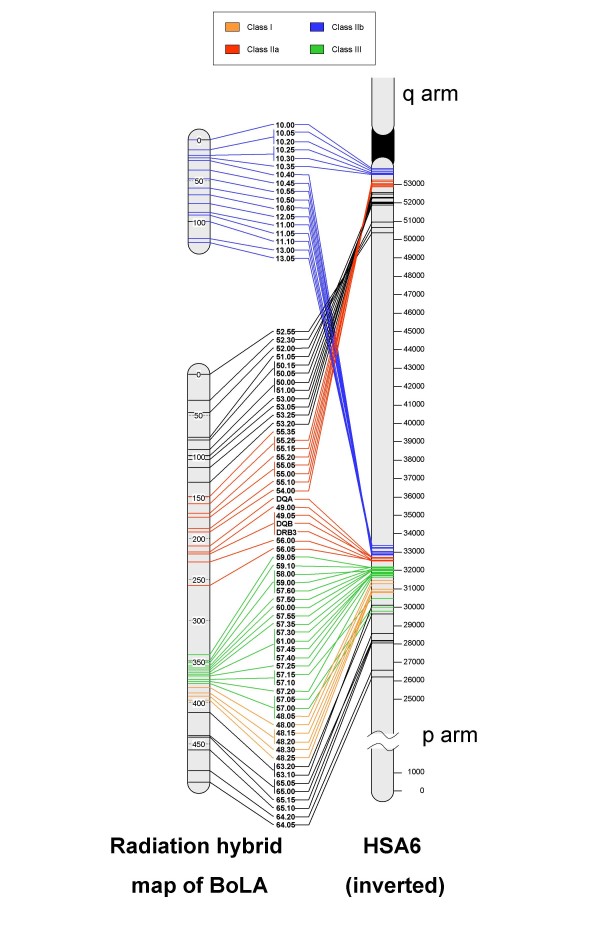
**Comparison of BoLA RH map with homologous human region**. Comparison of the high-resolution radiation hybrid map developed in this study with the corresponding region of human chromosome 6 (HSA6). The radiation hybrid map of BoLA is on the left, with units given as cR. Marker names to the right of this are in the order determined through RH mapping. A representative map of HSA6 (with inverted orientation) is located at the far right; connecting lines provide a comparative view of the marker order between the RH map and HSA6. The centromere is represented as a black region in between the two arms, which are labeled "q arm" and "p arm", and distances are given in Kb.

The physical order of all markers on the RH map was compared with both the Btau_3.1 and 4.0 assemblies and the NCBI36 assembly of the human genome sequence. Results of these comparisons are shown in Figures 1 and 2 respectively.

#### Comparison of RH map and Btau_3.1 assembly

The organization of the four scaffolds and the 17 markers of the IIb region are essentially the same between the RH map and Btau_3.1 assembly. Exceptions are the inverted order of markers 10.50 and 10.55 and markers 11.00, 11.05, and 11.10 in the Btau_3.1 assembly relative to the RH map.

All markers from the extended class IIa region, with the exceptions of 52.00, 52.30, and 52.55, are placed more distally in the 3.1 assembly than in the RH map but with similar marker order. Marker order within the class IIa region is also in overall agreement between the RH map and 3.1 assembly with the single exception that class IIa markers 49.00 and 49.05, which map together on the RH map, are separated in the 3.1 assembly: marker 49.05 is placed within a group that includes class III markers and has likely been misplaced in the 3.1 assembly. Both scaffold and marker orders in this group are inconsistent with those identified by the RH map. The order of 57.05, 57.00, and all scaffold 48 class III markers are in agreement in both the Btau_3.1 assembly and the RH map, although the placements differ, with the RH map assigning these markers to a more distal location on chromosome 23. Finally, overall consistency was observed in marker orders within class I scaffolds 63, 65, and 64; nevertheless, scaffold 63 appears to be erroneously placed more proximal to scaffold 65 in the Btau_3.1 assembly.

Two markers, 55.35 and 59.10, were not assigned to BTA23 in the Btau_3.1 assembly although they are clearly present within BoLA based on analysis of the RH mapping data. Marker 55.35 is chromosomally unassigned within the 3.1 assembly, although analysis using Ensembl BLAST [27] returned an alignment with BTA23 at position 28243 Kb – clearly distal to the other markers of BoLA IIa. In contrast, the RH map shows 55.35 localized to the expected location near other markers from scaffold 55. The RH map has placed marker 59.10 at a predictable location near other markers from scaffold 59, while BLAST comparison against the Btau_3.1 assembly produced no significant alignment to 59.10.

#### Comparison of RH map and Btau_4.0 assembly

The IIb region of BoLA shows good agreement between the Btau_4.0 assembly and RH map, with the exception of an inverted segment encompassing three markers: 11.00, 11.05, and 11.10. This same inverted order was also observed in the comparison between Btau_3.1 and the RH map. Analysis of the positions of these three markers in an independent, finished assembly of BoLA IIb [28] supported the Btau_4.0 gene order and indicates that the discordancy is due to the imprecision of the 12,000_rad _RH panel to resolve the order of these closely linked markers. Marker order in BoLA IIb did not change from Btau_3.1 to Btau_4.0, but marker locations shifted ~2300 Kb telomeric on the chromosome in version 4.0 for all IIb markers except 13.00 and 13.05.

Class IIa, III and I marker order is very similar across the Btau_4.0 assembly of BoLA and RH map as is the order of markers in the extended IIa and I regions (Figure 1). Eleven markers located in two regions show some minor discordancy in gene order between the two maps. These include markers 51.05, 53.25, 55.15, 55.10, and 55.35 from BoLA IIa, and markers 59.05, 58.00, 57.50, 57.35, 57.20, and 48.00 from BoLA I. In most cases the difference in marker position is negligible but we were able to independently assess gene order for markers 59.05, 58.00, 57.50, 57.35, 57.20, and 48.00 in skimmed (2×) sequences of overlapping BAC clones (Projects VUAA-VUBO, Human Genome Sequencing Center, Baylor College of Medicine). This analysis determined that the distribution of markers in the BAC skims is consistent with the gene order predicted by Btau_4.0. Again, the few discordancies in gene order appear to be due to imprecise resolution of very closely linked markers in the RH panel limitations.

The only likely misassembly in Btau_4.0 detected in this study is the class IIa marker 55.35, which is positioned at the boundary of the IIa – III region in the assembly compared with a position in the centromeric region of IIa in the RH map. As a whole, the IIa/III/I region of BoLA is shifted ~3900 Kb telomeric in the Btau_4.0 assembly compared with the Btau_3.1 assembly.

Three markers, 10.50, 49.05 and 59.10, are all present on the RH map but missing in the BTA23 sequence and in the chromosomally unassigned contigs of the Btau_4.0 assembly. BLAST analysis of the marker sequences against the NCBI trace archives identified all three marker sequences, revealing that the Btau_4.0 assembly is missing at least three segments of BoLA DNA.

#### Comparison of RH map and human genome

**Figure 3:**
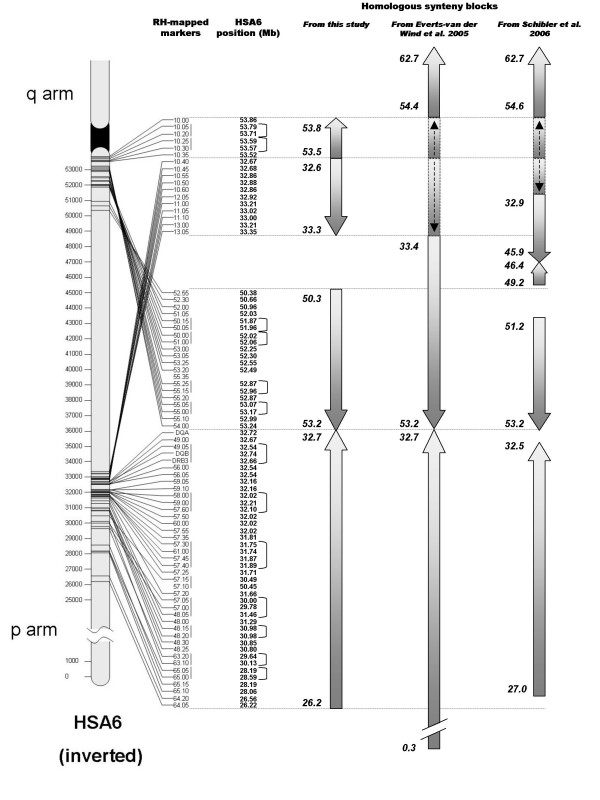
**Homologous synteny blocks in cattle and human**. Details about human homologs identified for RH-mapped markers used in this study. To the left, the BoLA RH-mapped markers are presented in their homologous HLA positions, with the corresponding megabase position of the human homolog listed to the right. Brackets indicate markers clustered by the RH analysis. These clustered markers have been assigned to the same cR position and therefore can be flipped with equal likelihood; as a result, caution should be exercised when considering these markers as part of an inversion. To the right, arrows indicate locations and orientations of homologous synteny blocks in relation to human, determined by this study, [14], and [29]. Proposed extensions of HSBs are represented as lighter-colored blocks with dotted edges; orientation is indicated by arrows within the blocks.

Comparative analysis of the RH map of BoLA and the corresponding sequence information for HSA6p identified four homologous synteny blocks (HSB) (Figures 2 and 3). Two HSBs are located in the IIb region of BoLA and correspond to sequences at 32.6–33.3 Mb and 53.5–53.8 Mb on HSA6. These HSBs represent the regions of HSA6p12 and 6p21 that are contiguous in the bovine genome. The other two HSBs correspond to the IIa/III/I region of BoLA and to sequences 26.2–32.7 Mb and 50.3–53.2 Mb on HSA6 as previously described [14,29]. The positions and boundaries of these blocks were compared with those described in previous studies ([14,29], Figure 3). These studies also described a HSB corresponding to the 54.4–62.7 Mb [14] or 54.6–62.7 Mb [29] sequence segment of HSA6 proximal to the centromere. Based on our results, this region of conserved synteny may be extended to include sequences at 53.5–53.8 Mb on HSA6p as an extension of this same HSB. The previous studies also identified a HSB corresponding to sequence at 33.4–53.2 Mb [14] or 32.9–45.9 Mb on HSA6p [29]; the lower boundary of this block can be extended to position 32.6 Mb on HSA6p based on our results. The remaining two HSB found in this study, corresponding to sequences 50.3–53.2 Mb and 26.2–32.7 Mb on HSA6p, confirm the results of Schibler et al [29] and Everts-van der Wind et al [14].

Although coding sequences were not used as sources of STS markers in this study, the stringent requirements used to select STS for RH mapping enabled us to reliably identify homologous loci within HSA6 for all but two of the bovine STS markers mapped in this study. These conserved STS markers are incorporated into Figure 2 to integrate the RH map with the sequence-based map of HLA. The two markers that could not be located to HLA were marker 55.35, which returned no significant alignments to the human sequence, and marker 57.10, which returned multiple alignments to sequences in the HLA class I region. Marker 57.10 may be located in a region of HLA that has undergone an expansion not observed in the bovine MHC, thereby making it difficult to identify the location of the orthologous marker on HSA6.

## Discussion

### Radiation hybrid mapping of BoLA

The generation of a high-resolution, physically-ordered radiation hybrid map of BoLA provides an independent test of the order and arrangement of markers and scaffolds within the genome sequence assembly. This validation is critical for future studies involving the BoLA region, such as those that seek to identify sequence features (e.g. conserved functional elements) or clarify the evolution of this region. Importantly, the RH map can be used to facilitate comparisons of the region with other species – particularly helpful when the accuracy of an assembly is unknown or uncertain. The relatively high radiation dose used to construct the 12,000_rad _RH panel used in this study generally provided a high resolution of marker order for evaluation of sequence assemblies but caution should be exercised when relying strictly upon RH mapping to order very closely spaced markers.

### Comparative mapping

#### Comparison of RH map and Btau_3.1 assembly

Discrepancies identified between the RH map described here and the version 3.1 assembly of the bovine genome sequence highlight major potential errors in the assembly. Although both maps are in general agreement in the order of markers for the BoLA IIb region, the RH mapping data do not support the assembly of much of the IIa/III/I region, as indicated by the many inconsistencies observed between the RH map and sequence assembly (Figure 1).

The RH map compared to the Btau_3.1 map is in general agreement in the BoLA IIb region (~4579–5363 Kb sequence segment of BTA23) but is far less concordant for the IIa/III/I region. The arrangements of at least six blocks of sequence (~19635–20172 Kb, ~21650–21284 Kb, ~21595–21998 Kb, ~23268–23824 Kb, ~24069–24889 Kb, and ~25586–25667 Kb sequence segments in Btau_3.1 are in disagreement with map order as determined by RH mapping and indicates much misassembly of BoLA sequence in the Btau_3.1 assembly.

#### Comparison of RH map and Btau_4.0 assembly

The version Btau_4.0 assembly of the bovine genome is generally concurrent with the RH map of BoLA, demonstrating the improved reliability of the new assembly within this region. The order of markers across most of the BoLA regions in the version 4.0 assembly is in complete agreement with the RH map, with a few minor exceptions: namely, a small inversion in marker order involving markers 11.00–11.05–11.10 and some discordancy in the order of a few markers in BoLA IIa and BoLA I, all of which is attributed to insufficient power of the 12,000_rad _RH panel to resolve markers that are within a few Kb of each other.

#### Comparison of RH map and human genome

The development of a high resolution radiation hybrid map of BoLA allows for reliable multispecies comparisons of the MHC despite the absence of an accurate and consistent genome sequence assembly. The comparative marker order between BoLA and HSA6, when adjusted for the ruminant inversion (Figure 2), demonstrates a remarkable conservation of gene content and order between cattle and humans. Several rearrangements in marker order are observed between the maps of the two species and these regions are prime candidates for further study to improve our understanding the evolution of the mammalian MHC.

## Conclusion

The findings of this study provide a high resolution physically ordered radiation hybrid map of the BoLA region encompassing 80 markers. Comparisons with the Btau_3.1 assembly of the bovine genome sequence shed light on a number of discrepancies, indicating that caution should be used when interpreting results and drawing conclusions based on this assembly. The Btau_4.0 assembly of the bovine genome shows far better agreement with the RH map; the fact that this assembly can be verified in such a way is indicative of its reliability. As such, this assembly will be valuable for future studies in which assembly accuracy is paramount, such as searches for conserved elements or interpretation of evolutionary histories. Additionally, the resolution and reliability of the RH map enabled comparisons with the human genome that help to refine boundaries of HSBs. This map will prove useful for future studies of this region of the cattle genome as it gives clarification regarding the structure and organization of BoLA and highlights potential inaccuracies in the Btau_3.1 assembly.

## Methods

### Primer design and optimization

Markers for genotyping the 12,000_rad _RH panel were identified from the Btau_2.0 assembly of the cattle sequence based on BLAST alignments to coding sequences from the human MHC (NCBI build 36). Assembly scaffolds of bovine DNA containing human MHC homologues were masked for repetitive DNA with RepeatMasker [30] and unique PCR primers were designed at ~50 kb intervals across the entire version 2.0 assembly of BoLA regions. Primers were designed using the Primer3 program [31] and were further evaluated against the Btau_3.1 assembly to assure that unique, highly conserved sequence tracts were selected for primer design. Previously published primers for BoLA class II *DQA*, *DQB*, and *DRB3 *[32,33] were also used for genotyping the RH panel. Detailed primer information is presented in Additional file 1.

### Genotyping primers on 12000_rad _RH panel

PCR typings were performed in duplicate in 10-μl reaction volumes containing 50 ng DNA, 1× buffer (Sigma Aldrich), 0.3 pmol of each primer, 0.2 mM dNTPs, 1.5 mM MgCl_2_, and 0.25 U JumpStart REDTaq DNA polymerase (Sigma Aldrich). Amplification parameters included an initial 30-sec denaturation at 94°C; 1 cycle of 94°C for 30 sec, 60°C for 30 sec, and 72°C for 30 sec; followed by 35 cycles of 30 sec at 94°C (denaturing), 30 sec at optimized annealing temperature (ranging from 56°C to 64°C), and 30 sec at 72°C (extension); ending with a final extension for 5 min at 72°C. Amplification products were resolved by electrophoresis in 1.0% agarose gels containing 0.36 μg/ml ethidium bromide, photographed and manually scored for the presence of the bovine-specific amplicons. Bovine, hamster and negative control (no DNA) samples were included in each amplification experiment. All samples were independently genotyped at least twice using this protocol.

### Computation of RH maps

Radiation hybrid maps were constructed with the software package *rh_tsp_map *[34,35], which utilizes Qsopt [36] and CONCORDE [37] to construct robust RH maps. Maps were built in such a way as to optimize maximum likelihood (MLE) criterion, as described previously [38]. Specifically, pairwise LOD scores were calculated and linkage groups were identified using the maximum LODs that still allowed the formation of single groups for the two main BoLA regions: LOD thresholds of 20.0 and 11.0 for the regions containing BoLA IIb and BoLA IIa, III, and I, respectively. Framework maps were constructed using the program *frame_markers *and comprehensive maps were generated with CONCORDE based on five different criteria (base MLE, extended MLE, normalized MLE, normalized OCB, and weighted OCB). The framework and comprehensive maps were then compared using the program compare_frame_script, and markers found to be incongruous between the two maps were removed from the framework. This process was then repeated until there were no discrepancies between the different maps. Each map is an optimal marker order based on the MLE defining criteria; therefore, the maps are termed "MLE-consensus maps" rather than the more commonly used "framework map".

To test the robustness of the maps, the programs map_eval and flips were used to evaluate the position of each marker in relation to other markers. The program map_eval was used to calculate the difference in score between the best and second best positions for each marker; markers with a threshold of change below 0.5 were removed from the framework. The program flips was then used to determine the best local arrangement of markers by examining all possible permutations of marker order within a sliding window. Lastly, additional markers were added to the MLE-consensus maps using the program bin_script, with an upper threshold of 0.1, a lower threshold of 0.0, an upper gap threshold of 3, and a lower gap threshold of 1 to allow markers that map to identical positions to be put into a common bin.

### Construction of comparative maps

Maps were generated to indicate the positions of the RH-mapped markers in relation to the version 3.1 and 4.0 assemblies of the bovine genome [39] and to the human genome (NCBI build 36) of HLA. To identify conserved markers, the tract of sequence used for primer design was analyzed using the BLASTN option of the Ensembl BLASTview tool [27] against the version 3.1 assembly of the bovine genome with search sensitivity of "Near-exact matches", and against the human genome build 36 using a search sensitivity of "Distant homologies". BLAST alignment against Btau_4.0 was performed locally using linear scaffold data for each chromosome, available from [18], and the same primer sequence tracts used for the Btau_3.1 and human comparative maps.

An alignment result was used for comparative mapping with human only if the sequence was located on human chromosome 6p (HSA6p), which contains HLA; and had an E-value less than 10e-5. If these criteria were met, the position of the alignment result was used to map the RH-mapped marker relative to other markers already on the human map. One marker (10.25) did not have an E-value low enough to meet the second criteria but was not excluded from analysis because alignment results gave the expected location on HSA6. Marker 55.35 did not meet either criteria and was therefore removed from the comparative analysis. A third marker, 57.10, gave unreliable alignment results on HSA6 with relatively high E-values (≥ 3.2) and was also removed. All other bovine markers were mapped on HLA. Homologous synteny blocks (HSB) were determined according to the criteria outlined in [40].

## Authors' contributions

CLB-L synthesized and interpreted the data, constructed comparative maps, and drafted the manuscript. CPC designed primers and performed the RH map computation. KLF coordinated and performed RH typing. ALG-S and TR provided assistance with typing and ensured that all data met specific quality and reproducibility criteria. MC performed RH typing and established procedures to ensure data consistency. JEW provided the 12,000_rad _RH panel. LCS conceived of the study, participated in its design and coordination and helped to draft the manuscript. All authors read and approved the final manuscript.

## Supplementary Material

Additional file 1 **Primer information**. Detailed primer information for RH-mapped markers, including classification (I, IIa, IIb, III), position within Btau_3.1, primer sequences, product sizes, and annealing temperatures used. All PCR reactions were carried out using 1.5 mM MgCl_2_. *For *DQA *and *DQB *two forward primers were multiplexed and used with a single reverse primer. Click here for file

Additional file 2 **Marker information**. Centiray position, lod score, and frame/placed status for each marker. Note that markers labeled as "frame" are considered to be part of the MLE map. Markers 48.10 and 55.30 could not be assigned a discrete location and instead were tentatively localized to specific regions between two markers as indicated by the column labeled "binned between". Click here for file
