# Supplementary material for: A high resolution RH map of the bovine major histocompatibility complex
Source: BMC Genomics. 2009 Apr 24;10:182. doi: 10.1186/1471-2164-10-182 (PMC2682492; doi:10.1186/1471-2164-10-182)
Supplement: Additional file 1 — Primer information. Detailed primer information for RH-mapped markers, including classification (I, IIa, IIb, III), position within Btau_3.1, primer sequences, product sizes, and annealing temperatures used. All PCR reactions were carried out using 1.5 mM MgCl2. *For DQA and DQB two forward primers were multiplexed and used with a single reverse primer. [file 1471-2164-10-182-S1.doc]

**Additional file** 1: Primer information

| Marker | Class | Position (Btau_3.1) | Forward and Reverse Primers  5'--3' | Product size (bp) | Annealing temp (°C) | Reference |
| --- | --- | --- | --- | --- | --- | --- |
| 10.00 | IIb | 4578989 | F: TGTCTATCCTGTCCCAGAGCTA  R: CGATTTCTCTCATGCTTACCTG | 279 | 54 |  |
| 10.05 | IIb | 4650632 | F: GTGCCTACGTTACAGCTCATTC  R: CTCTCTTTTCTTGTTCCCAGGT | 262 | 56 |  |
| 10.20 | IIb | 4733362 | F: CTGTAACTATCGCCATCAGGAG  R: CTCAAGTAAGCTGCATCCATTC | 301 | 56 |  |
| 10.25 | IIb | 4826184 | F: ACAGCATGTGGGATGTTAGTTC  R: GCCTTCACACTTAGAGCAACAC | 256 | 56 |  |
| 10.30 | IIb | 4864347 | F: CAGAAAGGAAAGCTGTTGACAC  R: CAGTTTGAAGCCCATTACTCAG | 338 | 54 |  |
| 10.35 | IIb | 4932881 | F: AAATGCCTCTCCCTCTCAGTAG  R: TAAGGACACTGGTTTGTTCCTG | 312 | 54 |  |
| 10.40 | IIb | 4982555 | F: TCAAGCTCTACCTGTCCAAGAA  R: CATCTTCACTGTTGCTTTCCTC | 284 | 58 |  |
| 10.45 | IIb | 5022325 | F: AGTTTTCCAGGCTTCTTACAGG  R: GTACACTCACCCACGATGTCTT | 307 | 56 |  |
| 10.50 | IIb | no hit BTA23 | F: AAGTTGAAGACCACGACTCAGA  R: CTGTGAGGTCAATGTTCAGGAT | 250 | 56 |  |
| 10.55 | IIb | 5065052 | F: ATGAGTTAGGGAAGAAGGGTCA  R: TCACCGTCTATATCAAGCATCC | 286 | 56 |  |
| 10.60 | IIb | 5111328 | F: TCCCCTACCTCACCAGATAGTT  R: ACCCACTAGGAAGAAGGTTTCA | 301 | 54 |  |
| 11.00 | IIb | 5362858 | F: TAACCGCAGCAACTAGAGAGAG  R: GAATAGGCAGGTGGGAGAATAC | 345 | 56 |  |
| 11.05 | IIb | 5293903 | F: GTTTCACTCGGATGGAGATACA  R: AGGCAGGATGTAGAGACACTGA | 287 | 56 |  |
| 11.10 | IIb | 5278775 | F: GGACAATATCATCAGTGGGCTA  R: CAGGAGACTTGCGTAGAAGAGA | 263 | 56 |  |
| 12.00 | IIb | 5164294 | F: CAGCTAATGAGTATGCCTCCAG  R: CCCAGCTCTTTCTCTCTCTCTC | 342 | 56 |  |
| 12.05 | IIb | 5142876 | F: CTAAGGTGACAGATGTGGGTGT  R: AAAGCGAAGTAGGGTAGTGAGG | 345 | 56 |  |
| 13.00 | IIb | 7849817 | F: CTCACCGTTTTAAGAGCTTCCT  R: CTTTCCCATTAACCTGTTCCTC | 322 | 56 |  |
| 13.05 | IIb | 7897638 | F: CAAGCTCCACCACAGAACTAAC  R: ATCAGCTATCCCAGAGGAAGAG | 260 | 56 |  |
| 48.00 | I | 21723458 | F: ACCTGGATAGAGCTGACACAGA  R: GTTCCTCAGTAGTCTGGTGCTG | 317 | 60 |  |
| 48.05 | I | 21809878 | F: CCAGAGGGTTAGGTTTAGGATG  R: CTACCTCCCTTCAGCTTTCACT | 280 | 58 |  |
| 48.10 | I | 21855365 | F: CTTCATATAGGTTGGGGAGGAG  R: AGCACTTCGTGACTAGGAAACA | 290 | 58 |  |
| 48.15 | I | 21871079 | F: GGAAATAGGAACTGTGGGTCTC  R: CCTGAGATACGGGTGTTGATAA | 318 | 62 |  |
| 48.20 | I | 21910345 | F: AGAAAGAAGGAGACCCACAAAG  R: CTAGGCTTCCCAAACTGCTATT | 242 | 60 |  |
| 48.25 | I | 21998611 | F: AGACTGATGAATGGATGACCAG  R: TAAGGGCAGGGAAAAGAGATAG | 280 | 60 |  |
| 48.30 | I | 22023909 | F: CTTCCTTCCCATTACCTTTGTC  R: CCTAAATCCATCTCCTTTGAGG | 271 | 58 |  |
| 49.00 | IIa | 21338252 | F: GGAACCTCTATGGTGAGGAAAC  R: ATCTTCTGCCTTCTTCCAACTC | 278 | 56 |  |
| 49.05 | IIa | 24616715 | F: CCCTTTGATTTGGAGTAGGTTC  R: GAGATTGGACACTGAGGATTGA | 258 | 56 |  |
| 50.00 | IIa extended | 23337409 | F: GGTGAGCTTTCATCTGACTGTG  R: GGGTCTCTAAGGGTCTTCCACT | 165 | 62 |  |
| 50.05 | IIa extended | 23268559 | F: TTCTCAGACCCGTCCTTACAAT  R: GCAACCACTTTACCTGCTATCC | 173 | 58 |  |
| 50.15 | IIa extended | 24505218 | F: GCAAAGAATTGATTTCCACACA  R: AAACAGTCGGATGGCATTACTT | 166 | 58 |  |
| 51.00 | IIa extended | 23377033 | F: ATCCTACATGCGGGAGTTTCTA  R: GCATTTCTGAGTGCCATATGAA | 180 | 58 |  |
| 51.05 | IIa extended | 23349783 | F: ACTGGAGAGGCATGCATAGAAT  R: ACCCCAGTAAACACATCAGGAC | 164 | 58 |  |
| 52.00 | IIa extended | 20172530 | F: TTAACAAAGGATCAGAGCAGCA  R: AGGCAGTTGTATAGCGGTTGTT | 159 | 56 |  |
| 52.30 | IIa extended | 19898687 | F: AGGATTCCCAATCTTCTTGTGA  R: CATTCAAACGTGCATCAAACTT | 235 | 58 |  |
| 52.55 | IIa extended | 19635295 | F: ATGGCAATATTTGTTCCTGGAC  R: TTCCCCTTCAAGTGTATGGTCT | 158 | 58 |  |
| 53.00 | IIa extended | 23568398 | F: GGCTGTGACCCATCTTTAAAAC  R: GACAGTCTCTTTGCTCATGTGG | 192 | 58 |  |
| 53.05 | IIa extended | 23624735 | F: TTTGACATGACACACTTCACCA  R: AAGCATTCTTTGAAAGGACGAG | 200 | 58 |  |
| 53.20 | IIa extended | 23776105 | F: TGCTTGACTTTGGAGAGATGAA  R: TCTTGGCTAAAAACATCCACCT | 223 | 58 |  |
| 53.25 | IIa extended | 23824231 | F: CAGGAGGAACTCTAGCTCCAAA  R: AGTTCCTGACTTACGCGTCTTC | 186 | 58 |  |
| 54.00 | IIa | 20972602 | F: CTCCGAATACCCTGTTGACATA  R: CCTTCCACTTCCATACAGATCA | 288 | 58 |  |
| 55.00 | IIa | 20933440 | F: GTCACACAAATAGAGCAGCACA  R: CAGTCACAGGCTTAGATTTTGC | 251 | 58 |  |
| 55.05 | IIa | 20862483 | F: GCCATATTTCAGGGTCTCTAGG  R: CCAGCTTTGTTTCTTCTCACAC | 250 | 56 |  |
| 55.10 | IIa | 20814904 | F: AAGAGTGATGCAGACACGATCT  R: TCCTCAGTATAATCGCATGGTC | 238 | 56 |  |
| 55.15 | IIa | 20785235 | F: CTTCTGGAACTGCTTATCATGC  R: TGACAGTGCATACAGAGGTGAG | 314 | 58 |  |
| 55.20 | IIa | 20696883 | F: GACATGACTGAGGTGGTCCTAA  R: AGGCAGCAAAGTAATTCTCCTC | 286 | 58 |  |
| 55.25 | IIa | 20678140 | F: TGCCACCAAATACAACCTCTAC  R: GATGGTATCCTCTTCCATAGGC | 238 | 58 |  |
| 55.30 | IIa | 20626470 | F: AGTTCTTCCAGCTCTCCTCATC  R: GAATATCCTGCTTTCAGCCTCT | 321 | 58 |  |
| 55.35 | IIa | ChrUn | F: CGACATACAGAACCCATCCTTA  R: ACTTCTTTCCCTTTCAGTCCAG | 326 | 58 |  |
| 56.00 | IIa | 21464235 | F: GGTCTTAAAATCTGGGGACTTG  R: TCTCTGCCACTGTCCACTACTT | 246 | 56 |  |
| 56.05 | IIa | 21469205 | F: TATTCCCTCTGCCTTTTCTCTC  R: GAGCTTCTCACTCCATTCCTCT | 297 | 58 |  |
| 57.00 | III | 21672247 | F: ACTCTACCCAGCACTCTCAACA  R: GTCTCTGCCTCCTACCAAGACT | 316 | 56 |  |
| 57.05 | III | 21595247 | F: ACAGTCTCAGGAACATGAAGGA  R: AGAAGCACTCAGGTAGGGAAAG | 304 | 58 |  |
| 57.10 | III | 24889291 | F: CTAAGTTGCAGGGATAGCAGTG  R: CATTCGGCAGTTTCTACTGTTC | 328 | 62 |  |
| 57.15 | III | 24738326 | F: CGAGTTCAAGAAGTAGCCAATG  R: GGGATAACTGAAGAACGTGACA | 312 | 58 |  |
| 57.20 | III | 24694440 | F: GGACACAGGACACAGGTTAAAA  R: AAACACGAGTCTGGAAGCTACA | 296 | 58 |  |
| 57.25 | III | 24155407 | F: GCTTCCTTTAGTCTGCATCCTT  R: AAAGAGAGAGGGAGTTGGTGAG | 234 | 58 |  |
| 57.30 | III | 24097645 | F: AGATGTGACTATTGAGGCATGG  R: CCTTTCTAGGTGATCTGCTGTG | 309 | 58 |  |
| 57.35 | III | 24069307 | F: TTTTCTCCCTCTTCTAGGATGC  R: AGGAGGTTACATGACTCCCAGT | 261 | 56 |  |
| 57.40 | III | 24383873 | F: ATGATGACTCCAGGTGATTCTG  R: ACATACTAGGAAAAGGGGTGGA | 320 | 60 |  |
| 57.45 | III | 24364002 | F: CGACTCTAACTGGTCCTCACAG  R: CTTCCAGATCACGCTTTTGTAG | 313 | 58 |  |
| 57.50 | III | 24281359 | F: GCCAGCCATACCTACTCTCTCT  R: TCTTACTTGCTTTCAGCTCCAG | 299 | 56 |  |
| 57.55 | III | 24272913 | F: ACAGTGGTATTTGACTCCATGC  R: GAAAGCAGAGAGAGAGGAGAGG | 245 | 62 |  |
| 57.60 | III | 24182505 | F: GAGGACACACAAAAGTCACCAT  R: GACCACTTCCTGATCCAGTACA | 281 | 58 |  |
| 58.00 | III | 24237308 | F: CCTGAAACTCAGCCTTTTGC  R: GCCTGGAGAGTCAGACCAAG | 343 | 60 |  |
| 59.00 | III | 24465223 | F: TGTCTCATGCAGTCTTAACGTG  R: TCTAACCAGAAGCACACGAGTT | 327 | 58 |  |
| 59.05 | III | 24416257 | F: ATCCTCTCCTCTGCGACTTTAC  R: CTGGTATCCATGACCTCACACT | 272 | 58 |  |
| 59.10 | III | no hit BTA23 | F: CCTCATGGAGAGATGCAGTAAA  R: GTCACAGGAAAGGGAAAGAAAG | 266 | 58 |  |
| 60.00 | III | 24216318 | F: AGTCTCTCCCTTGTCTGTCCTC  R: GTTGTCAGGATGTAGGGGTTTT | 320 | 58 |  |
| 61.00 | III | 24122899 | F: TCCTCAGAAACCAGAGTGTGTT  R: GGCCTTGGTGAGTGAAACTT | 283 | 58 |  |
| 63.10 | I extended | 27064561 | F: GACCTCCATTGCCTCTGTTATC  R: CATATGGGGCAAATCATTTCTT | 154 | 58 |  |
| 63.20 | I extended | 26955742 | F: GGGAAGGAAGAGGACAGTCTTT  R: ACAAAGGTAGAGGAAGCGTGAG | 195 | 58 |  |
| 64.05 | I extended | 27269627 | F: TTAGCGAGTACTGGCAGAATGA  R: GACCGAAAGTGCCTCAGTAATC | 208 | 58 |  |
| 64.20 | I extended | 27568403 | F: CCATGCAGTGTTTAAAGGATCA  R: TCCATAAGCATCCAAGAGGAGT | 223 | 58 |  |
| 65.00 | I extended | 25667030 | F: GGCTTCTGCTCCTAAGTATGGT  R: CATAGGTTCTTGACTGCTTTGG | 240 | 58 |  |
| 65.05 | I extended | 25645406 | F: CACACTGATTGCACTGATATGG  R: AGACCTTCTGGGCTCATTAGAG | 304 | 58 |  |
| 65.10 | I extended | 25586620 | F: CACACGTACACACACATTCTCC  R: CTATGGTGATGCTGATCTGGTT | 279 | 58 |  |
| 65.15 | I extended | 25618331 | F: CCTGCTTATCTTCCTCCCTCTA  R: GTGGTAAATGGGAACTCCAGAT | 262 | 58 |  |
| DQA* | IIa | 21339287 | F: CTCCGACTCAGCTGACCACATTGG  F: CCTCAATTATCAGCTGACCACGTTGG  R: GACAGATGAGGGTGTTGGGCTG | 772-794 | 64 | [32], [33] |
| DQB* | IIa | 21320550 | F: TCCCCCGCAGAGGATTTCGTG  F: CTCCCCGCAGAGGATTTCGTG  R: CGCACTCACCTCGCCGCTGC | 289 | 62 | [32], [33] |
| DRB3 | IIa | 21376099 | F: ATCCTCTCTCTGCAGCACATTTCC  R: TTTAAATTCGCGCTCACCTCGCCGCT | 302 | 58 | [32], [33] |
