# Supplementary material for: A high resolution RH map of the bovine major histocompatibility complex
Source: BMC Genomics. 2009 Apr 24;10:182. doi: 10.1186/1471-2164-10-182 (PMC2682492; doi:10.1186/1471-2164-10-182)
Supplement: Additional file 2 — Marker information. Centiray position, lod score, and frame/placed status for each marker. Note that markers labeled as "frame" are considered to be part of the MLE map. Markers 48.10 and 55.30 could not be assigned a discrete location and instead were tentatively localized to specific regions between two markers as indicated by the column labeled "binned between". [file 1471-2164-10-182-S2.doc]

**Additional file 2: Marker information**

Upper RH group

| Marker | cR position | lod | frame/placed |
| --- | --- | --- | --- |
| 10.00 | 0.00 | 5.81 | frame |
| 10.20 | 12.26 | 5.81 | frame |
| 10.05 | 12.26 | 5.81 | frame |
| 10.25 | 19.16 | 5.49 | placed |
| 10.30 | 19.16 | 5.49 | placed |
| 10.35 | 22.54 | 2.58 | placed |
| 10.40 | 25.92 | 9.70 | frame |
| 10.45 | 36.62 | 2.21 | frame |
| 10.55 | 47.32 | 2.21 | frame |
| 10.50 | 59.40 | 2.63 | frame |
| 10.60 | 67.64 | 5.12 | frame |
| 12.05 | 77.68 | 5.35 | frame |
| 11.00 | 88.02 | 2.74 | placed |
| 11.05 | 91.40 | 2.72 | frame |
| 11.10 | 100.14 | 2.72 | frame |
| 13.00 | 120.49 | 0.87 | frame |
| 13.05 | 125.60 | 0.87 | frame |

Lower RH group

| Marker | cR position | lod | frame/placed | binned between |
| --- | --- | --- | --- | --- |
| 52.55 | 0.00 | 5.75 | frame |  |
| 52.30 | 32.10 | 3.76 | frame |  |
| 52.00 | 47.03 | 3.76 | frame |  |
| 51.05 | 76.97 | 0.33 | placed |  |
| 50.15 | 79.95 | 8.40 | frame |  |
| 50.05 | 79.95 | 8.40 | frame |  |
| 50.00 | 91.51 | 8.40 | frame |  |
| 51.00 | 91.51 | 8.40 | frame |  |
| 53.00 | 99.25 | 5.47 | placed |  |
| 53.05 | 104.04 | 8.81 | frame |  |
| 53.25 | 113.64 | 2.24 | frame |  |
| 53.20 | 131.31 | 4.17 | frame |  |
| 55.35 | 148.56 | 2.24 | frame |  |
| 55.25 | 157.74 | 6.51 | frame |  |
| 55.15 | 157.74 | 2.52 | placed |  |
| 55.20 | 169.48 | 6.51 | frame |  |
| 55.05 | 173.97 | 3.57 | frame |  |
| 55.00 | 173.97 | 3.57 | frame |  |
| 55.10 | 187.78 | 3.57 | frame |  |
| 54.00 | 192.27 | 4.87 | placed |  |
| DQA | 209.51 | 6.07 | frame |  |
| 49.00 | 216.70 | 6.07 | frame |  |
| 49.05 | 219.05 | 0.24 | placed |  |
| DRB3 | 219.05 | 0.42 | placed |  |
| DQB | 219.05 | 6.07 | frame |  |
| 56.00 | 228.65 | 4.42 | frame |  |
| 56.05 | 257.10 | 4.42 | frame |  |
| 59.05 | 341.42 | 2.79 | frame |  |
| 59.10 | 348.78 | 2.85 | placed |  |
| 58.00 | 351.07 | 2.85 | placed |  |
| 59.00 | 351.07 | 3.17 | placed |  |
| 57.60 | 351.07 | 2.85 | placed |  |
| 57.50 | 355.76 | 0.22 | placed |  |
| 60.00 | 358.10 | 2.79 | frame |  |
| 57.55 | 360.44 | 0.27 | placed |  |
| 57.35 | 362.84 | 5.14 | frame |  |
| 57.30 | 365.23 | 5.14 | frame |  |
| 61.00 | 365.23 | 5.14 | frame |  |
| 57.45 | 365.23 | 3.00 | placed |  |
| 57.40 | 365.23 | 5.14 | frame |  |
| 57.25 | 367.57 | 2.98 | placed |  |
| 57.15 | 372.26 | 2.98 | placed |  |
| 57.10 | 372.26 | 5.35 | frame |  |
| 57.20 | 374.55 | 2.98 | placed |  |
| 57.05 | 376.79 | 3.03 | placed |  |
| 57.00 | 376.79 | 3.03 | placed |  |
| 48.05 | 376.79 | 3.03 | placed |  |
| 48.00 | 381.37 | 5.35 | frame |  |
| 48.15 | 388.25 | 5.37 | frame |  |
| 48.20 | 388.25 | 5.37 | frame |  |
| 48.30 | 392.65 | 0.50 | placed |  |
| 48.25 | 397.14 | 4.51 | frame |  |
| 63.20 | 411.57 | 4.51 | frame |  |
| 63.10 | 411.57 | 4.51 | frame |  |
| 65.05 | 440.18 | 8.72 | frame |  |
| 65.00 | 440.18 | 8.72 | frame |  |
| 65.15 | 442.47 | 2.14 | placed |  |
| 65.10 | 457.98 | 7.76 | frame |  |
| 64.20 | 483.34 | 1.86 | frame |  |
| 64.05 | 496.60 | 1.86 | frame |  |
| 48.10 | -- | -- | binned | [48.20 - 57.10] |
| 55.30 | -- | -- | binned | [55.25 - 53.25] |
